# Optimal target blood pressure in critically ill adult patients with vasodilatory shock: A systematic review and meta-analysis

**DOI:** 10.3389/fphys.2022.962670

**Published:** 2022-08-16

**Authors:** Hidero Yoshimoto, Satoshi Fukui, Koki Higashio, Akira Endo, Akira Takasu, Kazuma Yamakawa

**Affiliations:** ^1^ Department of Emergency and Critical Care Medicine, Osaka Medical and Pharmaceutical University, Takatsuki, Osaka, Japan; ^2^ Department of Surgery, Osaka Medical and Pharmaceutical University, Takatsuki, Osaka, Japan; ^3^ Faculty of Medicine, Osaka Medical and Pharmaceutical University, Takatsuki, Osaka, Japan; ^4^ Trauma and Acute Critical Care Center, Tokyo Medical and Dental University Hospital, Bunkyo-ku, Tokyo, Japan

**Keywords:** mean arterial pressure, critical illness, hypotension, sepsis, vasoconstrictor agents

## Abstract

While the Surviving Sepsis Campaign guidelines recommend an initial target value of 65 mmHg as the mean arterial pressure (MAP) in patients with septic shock, the optimal MAP target for improving outcomes remains controversial. We performed a meta-analysis to evaluate the optimal MAP for patients with vasodilatory shock, which included three randomized controlled trials that recruited 3,357 patients. Between the lower (60–70 mmHg) and higher (>70 mmHg) MAP target groups, there was no significant difference in all-cause mortality (risk ratio [RR], 1.06; 95% confidence intervals [CI], 0.98–1.16) which was similar in patients with chronic hypertension (RR, 1.10; 95% CI, 0.98–1.24) and patients aged ≥65 years (RR, 1.10; 95% CI, 0.99–1.21). No significant difference in adverse events was observed between the different MAP groups (RR, 1.04; 95% CI, 0.87–1.24); however, supraventricular arrhythmia was significantly higher in the higher MAP group (RR, 1.73; 95% CI, 1.15–2.60). Renal replacement therapy was reduced in the higher MAP group of patients with chronic hypertension (RR, 0.83; 95% CI, 0.71–0.98). Though the higher MAP control did not improve the mortality rate, it may be beneficial in reducing renal replacement therapy in patients with chronic hypertension.

**Systematic review registration:** UMIN Clinical Trials Registry, identifier UMIN000042624

## 1 Introduction

In the treatment of critically ill patients, the optimization of systemic oxygen metabolism and local tissue perfusion of vital organs are important factors. Among the hemodynamic parameters, the mean arterial pressure (MAP) is a core indicator of the perfusion to vital organs and is used commonly in clinical practice. While the Surviving Sepsis Campaign guidelines recommend an initial MAP target value of 65 mmHg in patients with septic shock (Evans et al., 2021), we recognize that there is insufficient evidence supporting the use of this MAP value. The optimal MAP target for improving outcomes in patients with vasodilatory shock remains controversial (Corrêa et al., 2015; Leone et al., 2015; Russell, 2020). In patients with vasodilatory shock, catecholamines are used commonly to achieve and maintain the target blood pressure. However, the adverse events that are associated with catecholamines should be considered. Although maintaining normotensive blood pressure may play an important role in improving vital organ perfusion and preventing any organ damage that may be induced by vasodilatory shock (Fiorese Coimbra et al., 2019), adverse cardiovascular events as a consequence of catecholamine use, such as arrhythmia and ischemic events, are concerning, particularly in patients with chronic hypertension and in older adults (Xu et al., 2015). In a clinical setting, the net effect of the risk-benefit balance of catecholamine use can affect patient outcomes.

While the management of patients with vasodilatory shock, using a target MAP of ≥65 mmHg for initial resuscitation has been regarded commonly as good practice (Varpula et al., 2005; Maheshwari et al., 2018), two previous randomized controlled trials (RCTs), the SEPSISPAM (Asfar et al., 2014) and OVATION (Lamontagne et al., 2016) trials, that evaluated optimal MAP targets, showed no benefits in maintaining a high MAP using vasopressors even in patients aged 65 years or older. These RCTs and subsequent meta-analyses (Lamontagne et al., 2018; Hylands et al., 2017; D’Aragon et al., 2015) showed that management with higher MAP targets was not usually advantageous in patients with chronic hypertension. Contrarily, another RCT performed in the field of anesthesiology has suggested that management adjusted to individual resting blood pressure for high-risk surgical patients may reduce the risk of postoperative organ dysfunction, particularly renal dysfunction, when compared with the standard management of the minimal acceptable blood pressure (Futier et al., 2017). Thus, the optimal MAP target for management of vasodilatory shock, including septic shock, has not been determined because of the lack of sufficient evidence. In particular, it remains unclear whether maintaining a high MAP is associated with preventing organ damage due to adequate oxygenation or more adverse events with the use of vasopressors.

Although a newly available large RCT, the “65 Trial” by Lamontagne et al., has been published recently (Lamontagne et al., 2020), it has not yet been included in any systematic review or meta-analysis. We aimed to perform an updated systematic review and meta-analysis, including RCTs to evaluate the certainty of the evidence in the determination of the optimal MAP target for critically ill patients with vasodilatory shock.

## 2 Methods

### 2.1 Protocol registration

This study protocol was registered in the University Hospital Medical Information Network (UMIN) Clinical Trials Registry (Registration No. UMIN000042624). A systematic review and a meta-analysis were conducted according to the Preferred Reporting Items for Systematic Reviews and Meta-Analyses (PRISMA) statement (Page et al., 2021).

### 2.2 Focused review questions

The objective of this review was to determine the best clinical practice for hemodynamic stabilization in terms of optimal target blood pressure in critically ill adult patients with vasodilatory shock.

### 2.3 Types of studies

We only included RCTs that investigated optimal target blood pressure in critically ill adult patients with vasodilatory shock. Cohort studies, case-control studies, experimental animal studies, narrative reviews, correspondence, case reports, expert opinions, and editorials, were excluded.

### 2.4 Condition or domain studied

The study domain included adult patients with vasodilatory shock in critically ill settings.

### 2.5 Types of participants

We included studies with adult patients with vasodilatory shock caused by any underlying disease, such as sepsis, severe acute pancreatitis, postoperative complications, and other causes. We included studies that evaluated critically ill adult patients aged ≥16 years. Animal studies were excluded from this review.

### 2.6 Types of interventions and comparators

We included studies that compared the maintenance of the target MAP in two groups, a high-target MAP group and a low-target MAP group. The definitions of the actual target arterial pressure in both groups did not matter and may have varied across studies.

### 2.7 Types of outcome assessments

The primary outcome measure in the included studies was all-cause mortality. In cases in which multiple mortality outcomes were reported, the mortality outcome closest to the 28-days outcome was selected. We also assessed the outcome measures of intensive care unit and in-hospital mortality. As secondary outcome measures, the number of patients with organ dysfunction (respiratory and renal) and serious adverse events related to the study interventions was assessed.

### 2.8 Search strategy

We searched the following databases for relevant studies: MEDLINE (via PubMed), EMBASE, and Cochrane Central Register of Controlled Trials. We developed a search strategy using a combination of keywords and medical subject headings (MeSH)/EMTREE terms. The search strategy in MEDLINE was (“shock” [MeSH Terms] OR “vasopressor*” [Title/Abstract]) AND (“Blood Pressure” [MeSH Terms] OR “Blood Pressure” [Title/Abstract]) AND ((“randomized controlled trial” [Publication Type] OR “controlled clinical trial” [Publication Type] OR “randomized” [Title/Abstract] OR “placebo” [Title/Abstract] OR “clinical trials as topic” [MeSH Terms:noexp] OR “randomly” [Title/Abstract] OR “trial“ [Title]) NOT (“animals” [MeSH Terms] NOT ‘humans’ [MeSH Terms])). Our MEDLINE search strategy was adapted for searches in the two other databases (Fukui et al., 2021). We screened the reference lists of all the relevant studies for additional studies. No language or time restrictions were applied to the electronic searches.

### 2.9 Citation management and screening

The citations were stored and duplicates removed using EndNote software (Thomson Reuters, Toronto, Ontario, Canada). The studies were screened initially according to the title and abstract by two authors (SF, KH), independently, and those not meeting the criteria were discarded. Any disagreements were resolved by discussion and referral to a third author (KY) if necessary. After this initial stage, the full text of all the remaining studies was reviewed, and disagreements resolved in the same manner as in the initial screening. The Rayyan QCRI website (Ouzzani et al., 2016) was used in this screening process. The study selection process was documented using a PRISMA flow diagram.

### 2.10 Data extraction

Two authors (SF and KH) extracted the study characteristics from each included study, including data on the assessment of quality and the investigation of heterogeneity, and transferred that information into a study-specific format. A third author (HY) was consulted if necessary. Efforts were made to contact the authors of the primary studies to provide missing data where necessary.

### 2.11 Assessment of risk of bias

Two authors (SF and KH) assessed the risk of bias in the individual trials as well as the methodological quality of the articles, independently. Any disagreements were resolved by discussion, consensus, and the participation of a third author (HY) when necessary. To evaluate the risk of bias in individual RCTs, we used the revised uniform criteria of the Cochrane risk-of-bias tool for randomized trials ver. 2 (RoB 2) (Sterne et al., 2019). For each domain, we assigned an assessment value for the risk of bias as high, low, or some concerns.

### 2.12 Data synthesis

Statistical analyses were performed using Review Manager 5.4 software (Nordic Cochrane Center, Cochrane Collaboration) for each included trial. The risk ratios (RRs) were calculated with 95% confidence intervals (CIs) for all the outcome measures. We performed both a fixed-effects analysis using the Mantel–Haenszel method and a random-effects analysis using the DerSimonian and Laird estimator, reporting the most conservative summary estimate with the broadest CI.

### 2.13 Assessment of heterogeneity

Initially, we investigated the heterogeneity using a visual examination of the forest plots. The statistical heterogeneity was evaluated informally using the forest plots of the study estimates and more formally using the χ2 test (*p*-value < 0.1 = significant heterogeneity) and the I^2^ statistic (I^2^ >50% = significant heterogeneity). In addition, we performed a subgroup analysis to explore the potential sources of heterogeneity among trials. The analysis was performed using the following covariates: age, sex, disease severity, sepsis, existence of chronic hypertension, and targeted blood pressure.

### 2.14 Assessment of publication biases

We created funnel plots for mortality in which the log RRs were plotted against their standard errors and tested the symmetry of the funnel plots.

### 2.15 Rating of the certainty of evidence using the grading of recommendations assessment, development and evaluation (GRADE) approach

The risk of systematic errors (bias) and random errors was assessed, and the overall certainty of evidence was evaluated using the GRADE approach (Hsu et al., 2011).

## 3 Results

### 3.1 Literature search

**FIGURE 1:**
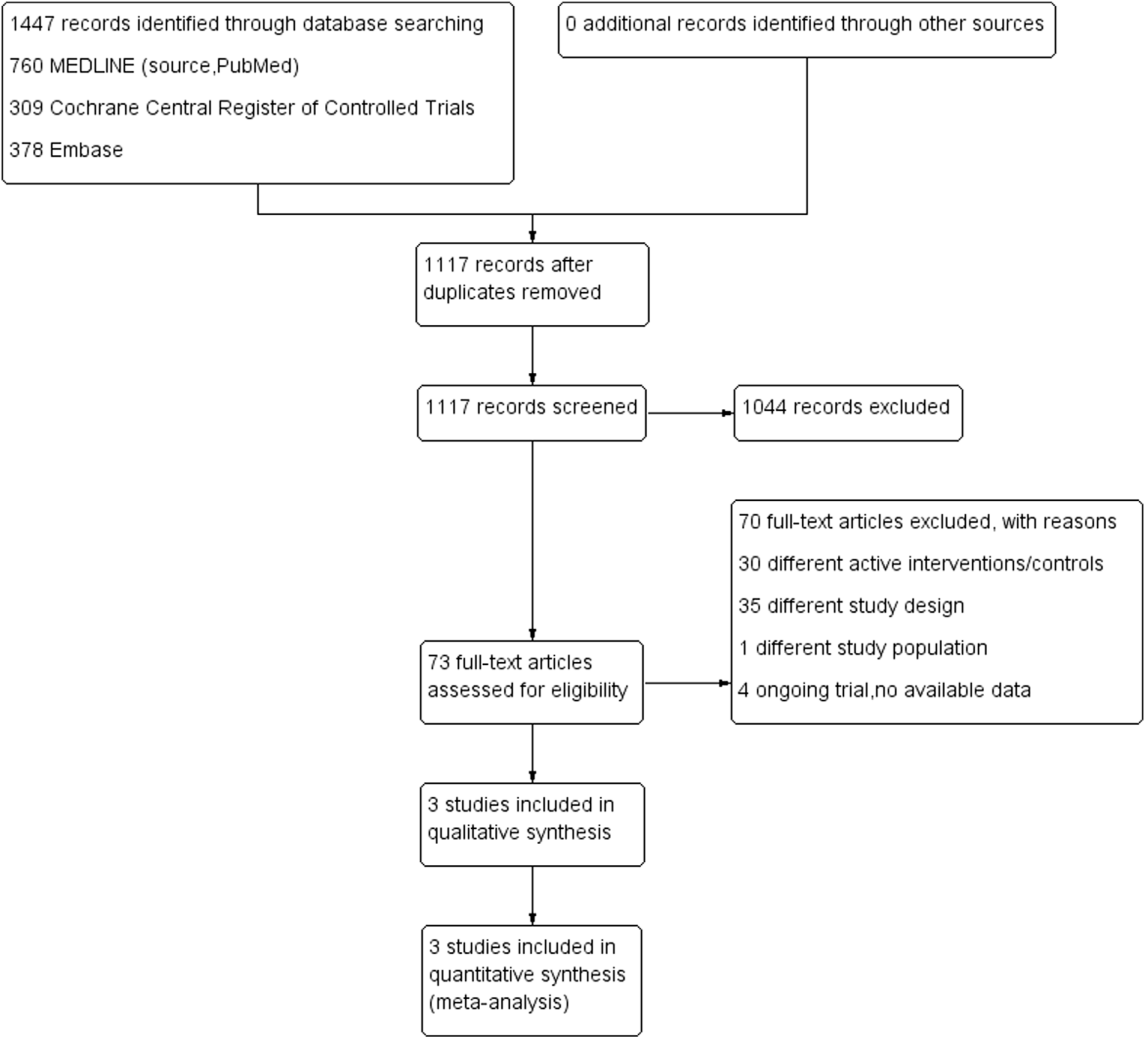
Preferred Reporting Items for Systematic Reviews and Meta-Analyses (PRISMA) chart: the identification and selection of trials for inclusion.

A PRISMA flow diagram of the selection process is shown in Figure 1. We identified 1447 records by searching the electronic databases. A total of 330 records were removed after deduplication. After screening according to titles and abstracts, 1044 records were not relevant and excluded. We assessed 73 full-text articles for their eligibility, and finally, we included three RCTs in this meta-analysis.

### 3.2 Baseline study characteristics

Table 1 shows the characteristics of the included studies. The trials recruited 3,357 patients aged ≥16 years from Canada, France, the United Kingdom, and the United States. The patients who received vasopressors had a lower MAP target of 60–70 mmHg or a higher MAP target of 70 mmHg or higher. In each trial, the most common cause of vasodilatory shock was sepsis. Among the trials, the target MAP was significantly lower in the lower MAP group than in the higher MAP group; however, the MAP values were higher than the predefined target in the lower MAP group. The most common vasopressor that was used was norepinephrine, which was infused for a longer period of time in the higher MAP target group. No clinical differences were observed in the values of fluid administration and urine output between the trials.

**TABLE 1:** Characteristics of the included randomized controlled trials.

Study	SEPSISPAM	OVATION	The 65 trial
Study venue	France (29)	United States (1), Canada (10)	United Kingdom (65)
Patient, n	776	118	2,583
Age, year	Lower MAP target: 65 ± 15	Lower MAP target: 66 ± 13	Permissive (lower MAP): 75.2 (70.4–80.5)
Mean (SD) or Median (IQR)	Higher MAP target: 65 ± 13	Higher MAP target: 63 ± 13	Usual care (higher MAP): 74.8 (70.1–80.8)
Sex, male (%)	Lower MAP target: 250 (64.4)	Lower MAP target: 31 (51.7)	Lower MAP target: 696 (57.2)
	Higher MAP target: 267 (68.8)	Higher MAP target: 33 (56.9)	Higher MAP target: 692 (55.8)
Inclusion criteria	Age ≥18 years, septic shock within less than 6 h, vasopressor infusion rate ≧0.1 μg/kg/min	Age ≥16 years old, vasodilatory shock regardless of admission diagnosis, vasopressor therapy for at least 6 h	Age ≥65 years, vasopressor infusion started for vasodilatory within prior 6 h and continued for 6 h or more
Exclusion criteria	Legally protected adults, not affiliated with a healthcare system	Receiving vasopressors for more than 24 h, expected to die within 48 h, or required vasopressors for unrelated reasons. Hypotension due to overt cardiogenic, hemorrhagic, or neurogenic shock or after cardiac surgery	Using vasopressors only for bleeding, acute ventricular failure, or post-cardiopulmonary bypass vasoplegia; undergoing treatment for brain or spinal cord injury; perceived imminent death; prior enrollment in the 65 trial
	Participation in another interventional trial, decision not to resuscitate		
Intervention	Lower MAP target: 65–70 mmHg	Lower MAP target: 60–65 mmHg	Permissive hypotension (lower MAP): 60–65 mmHg
Control	Higher MAP target: 80–85 mmHg	Higher MAP target: 75–80 mmHg	Usual care (higher MAP)
			The discretion of clinicians
Target MAP, mean (SD) or Median (IQR)	Lower MAP target: primarily between 70 and 75 mmHg	Lower MAP target: 70 ± 5	Lower MAP target: 66.7 (64.5–69.8)
	Higher MAP target: primarily between 85 and 90 mmHg	Higher MAP target: 79 ± 5	Higher MAP target: 72.6 (69.4–76.5)
Severity of illness score, mean (SD)	SAPS II	APACHE II score	APACHE II score
	Lower MAP target: 57.2 ± 16.2	Lower MAP target: 24 ± 8	Lower MAP target: 20.9 ± 6.5
	Higher MAP target: 56.1 ± 15.5	Higher MAP target: 25 ± 6	Higher MAP target: 20.6 ± 6.1
Age ≥65 years	415 (53.5)	65 (55.1)	2,463 (95.4)
Chronic arterial hypertension, n (%)	340 (43.8)	53 (44.9)	1,187 (46.0)
Sepsis, n (%)	776 (100)	83 (70.3)	1,917 (78.1)
Primary outcome	28-days mortality	ICU, hospital, 28-days and 6-months mortality	90-days mortality
Secondary outcome	90-days mortality	Persistent organ dysfunction	ICU, Acute hospital mortality
	Serious adverse events	Adverse events	Advanced respiratory and renal support
			Serious adverse events
Flow-up	6-months	90-days	1-year

### 3.3 Risk of bias

**FIGURE 2:**
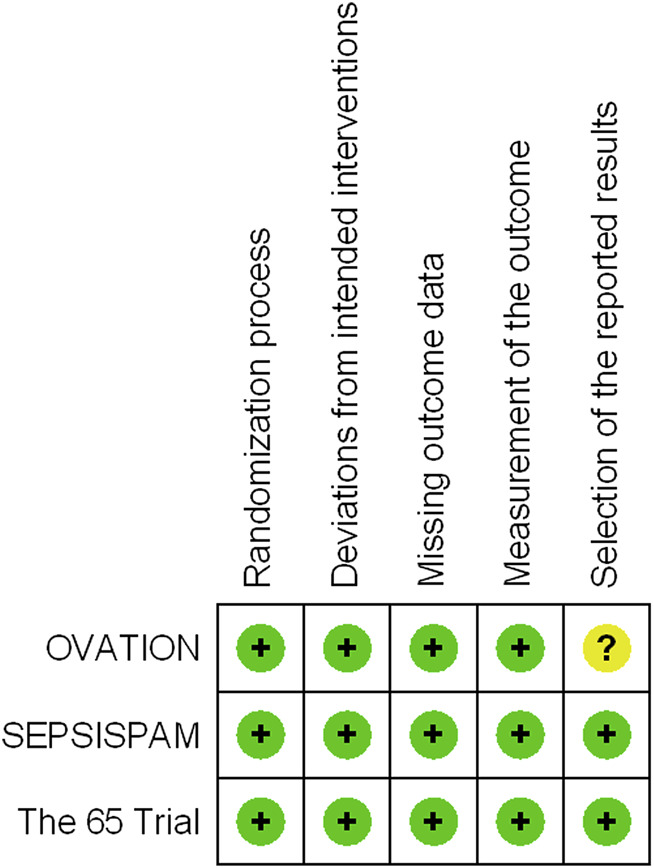
Risk of bias summary: a review of the authors’ assessments of each risk-of-bias item for each included trial.

We evaluated the quality of the three trials using the Cochrane RoB 2 tool (Figure 2). All the trials demonstrated a low risk of bias for all the categories. Among the three trials, the allocation sequence was concealed and randomized, missing outcome data was 0–5.4% of the total reported data, intention-to-treat was conducted, assessors of the assigned interventions were concealed, and the primary outcome was reported in its entirety. With regard to deviations from the intended intervention, all three trials were open-label RCTs. Due to the nature of the primary outcome (mortality), we assessed the risk of deviation from the intended intervention to be low. To the best of our knowledge, the protocol for OVATION (Lamontagne et al., 2016) was not available, but we considered that the outcome was mortality, and the risk of bias in the selection of the reported results was low.

### 3.4 Mortality

**FIGURE 3:**
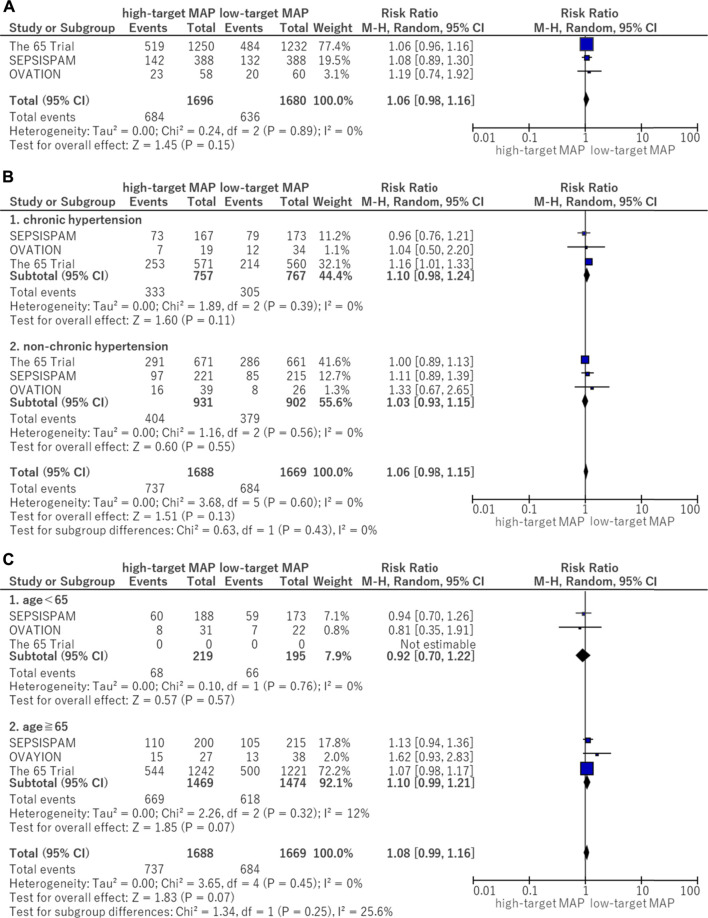
Forest plot of the comparison of a higher MAP versus lower MAP: Mortality. **(A)** All-cause mortality (closest to 28 days). **(B)** Mortality for patients with chronic hypertension. **(C)** Mortality for age <65 and ≧ 65. CI, confidence interval; M-H, Mantel–Haenszel; MAP, mean arterial pressure.

The forest plots for all-cause mortality are illustrated in Figure 3. There was no significant difference in all-cause mortality between the higher and lower MAP target groups (RR, 1.06; 95% CI, 0.98–1.16) (Figure 3A). Between the patients with and without chronic hypertension, no significant difference was observed in mortality between the MAP groups. A similar lack of significant difference was observed in the results between the higher and lower MAP groups in patients with chronic hypertension (RR, 1.10; 95% CI, 0.98–1.24) (Figure 3B). There were no significant differences in mortality in the context of patients aged 65 years and older (RR, 1.10; 95% CI, 0.99–1.21) or for those under 65 years of age (RR, 0.92; 95% CI, 0.70–1.22) between the MAP groups (Figure 3C).

The pooled mortality with vasodilatory shock due to sepsis was higher in the higher MAP target group than in the lower MAP target group (RR, 1.12; 95% CI, 1.02–1.23). Although the mortality rate of vasodilatory shock without sepsis was similar between the MAP groups, significant heterogeneity was detected between the subgroups with and without sepsis (I^2^ statistic = 52%).

### 3.5 Use of advanced respiratory support

**FIGURE 4:**
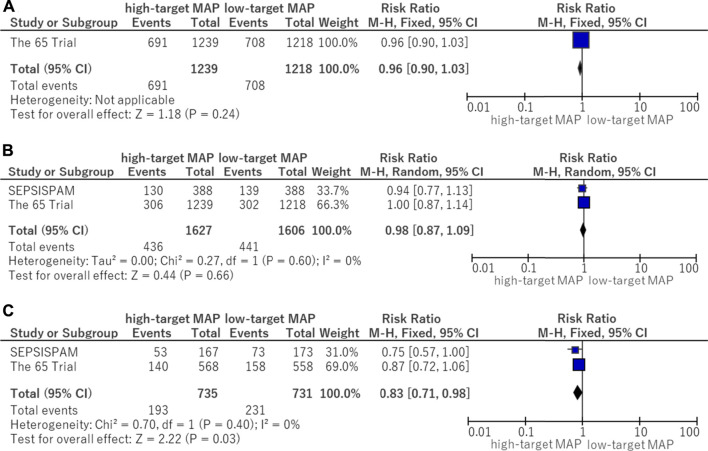
Forest plot of the comparison of a higher MAP versus lower MAP: secondary outcomes. **(A)** Use of advanced respiratory support. **(B)** Use of renal replacement therapy. **(C)** Use of renal replacement therapy for patients with chronic hypertension. CI, confidence interval; M-H, Mantel–Haenszel; MAP, mean arterial pressure.

In this analysis of available data, there was no significant difference in the use of advanced respiratory support (RR, 0.96; 95% CI, 0.90–1.03) between the higher MAP and lower MAP target groups (Figure 4A).

### 3.6 Use of renal replacement therapy

There was no significant difference in the use of renal replacement therapy (RR, 0.98; 95% CI, 0.87–1.09) between the higher MAP and lower MAP target groups (Figure 4B). Although no significant difference in the use of renal replacement therapy was found in patients without chronic hypertension, renal replacement therapy was reduced in those with chronic hypertension who were assigned to the higher target MAP group (RR, 0.83; 95% CI, 0.71–0.98) (Figure 4C).

### 3.7 Serious adverse events

**FIGURE 5:**
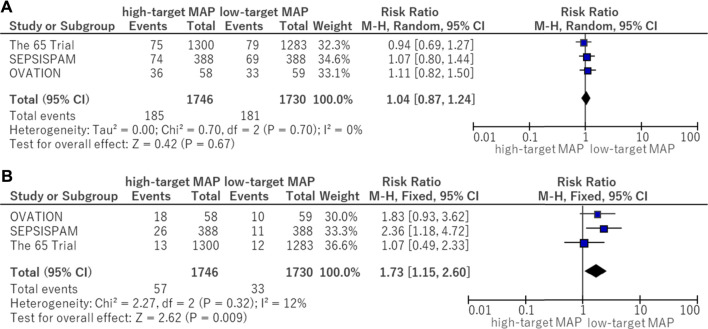
Forest plot of the comparison of a higher MAP versus lower MAP: adverse events. **(A)** Serious adverse events. **(B)** Supraventricular arrhythmia. CI, confidence interval; M-H, Mantel–Haenszel; MAP, mean arterial pressure.

In the subgroup analysis of serious adverse events, the following adverse events reported in the OVATION trial were excluded: suspected bowel ischemia, gastric feeding intolerance, and venous thromboembolic events because these events were not considered to be serious adverse events. There was no significant difference (RR, 1.04; 95% CI, 0.87–1.24) between the higher and lower MAP target groups (Figure 5A). The risk of supraventricular arrhythmia was significantly higher in the high MAP group (RR, 1.73; 95% CI, 1.15–2.60) (Figure 5B).

### 3.8 Publication biases

**FIGURE 6:**
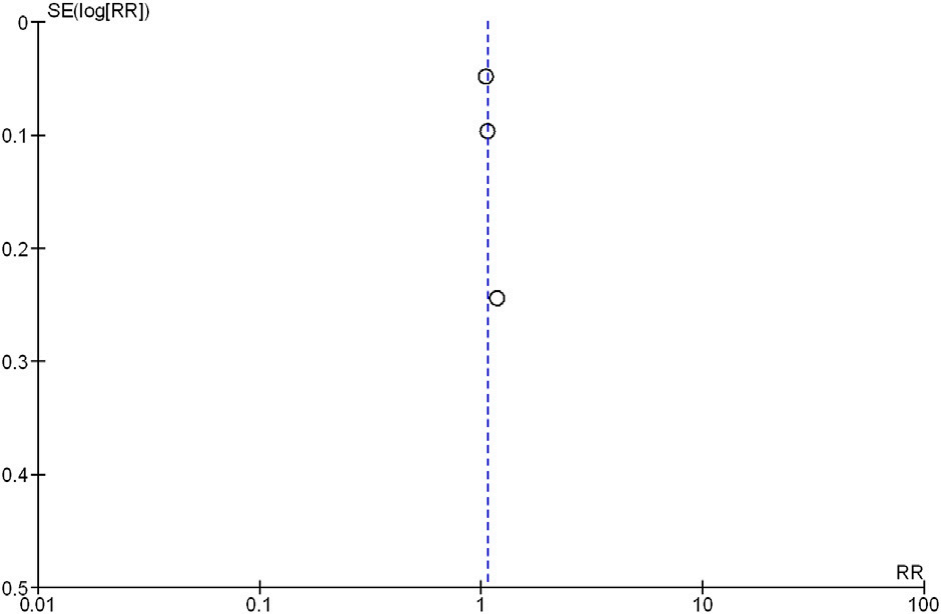
Funnel plot of the comparison of a high-target MAP versus low-target MAP: all-cause mortality (closest to 28 days). SE, standard error; RR, risk ratio.

The Deek’s funnel plot test showed no evidence of publication bias (Figure 6).

### 3.9 Certainty of evidence using the GRADE approach

We summarized the main findings, including the certainty of evidence for each outcome in Table 2. After evaluating each outcome according to the assessment of five factors for downgrading the certainty of evidence, we performed no downgrading and assessed the certainty of evidence to be high for the critical, important outcomes used in this analysis.

**TABLE 2:** GRADE evidence profile.

Certainty assessment							No. of patients		Effect			
No. of studies	Study design	Risk of bias	Inconsistency	Indirectness	Imprecision	Other considerations	Lower MAP target	Higher MAP target	Relative	Absolute	Certainty	Importance
All-cause mortality (follow up: closest to 28 days)												
3	Randomized trials	Not serious	Not serious	Not serious	Not serious	None	636/1,680 (37.9%)	684/1,696 (40.3%)	**RR** 1.06 (0.98–1.16)	**25 fewer per 1,000** (from -8 more to 58 fewer)	⊕⊕⊕⊕ High	CRITICAL
Mortality for patients with chronic hypertension												
3	Randomized trials	Not serious	Not serious	Not serious	Not serious	None	305/767 (39.8%)	333/757 (44.0%)	**RR** 1.10 (0.98–1.24)	**42 more per 1,000** (from -7 fewer to 92 more)	⊕⊕⊕⊕ High	CRITICAL
Mortality for age 65 and older												
3	Randomized trials	Not serious	Not serious	Not serious	Not serious	None	618/1,474 (41.9%)	669/1,469 (45.5%)	**RR** 1.10 (0.99–1.21)	**36 more per 1,000** (from 0 more to 72 more)	⊕⊕⊕⊕ High	CRITICAL
The use of advanced respiratory support												
1	Randomized trials	Not serious	Not serious	Not serious	Not serious	None	708/1,218 (58.1%)	691/1,239 (55.8%)	**RR** 0.96 (0.90–1.03)	**-24 more per 1,000** (from -63 more to 16 more)	⊕⊕⊕⊕ High	IMPORTANT
The use of renal replacement therapy												
2	Randomized trials	Not serious	Not serious	Not serious	Not serious	None	441/1,606 (27.5%)	436/1,627 (26.8%)	**RR** 0.98 (0.87–1.09)	**-7 more per 1,000** (from -37 more to 24 more)	⊕⊕⊕⊕ High	IMPORTANT
Serious adverse events												
3	Randomized trials	Not serious	Not serious	Not serious	Not serious	None	181/1,730 (10.5%)	185/1,746 (10.6%)	**RR** 1.04 (0.87–1.24)	**1 more per 1,000** (from -19 more to 22 more)	⊕⊕⊕⊕ High	IMPORTANT

Abbreviations: GRADE, grading of recommendations assessment, Development, and Evaluation; MAP, mean arterial pressure; RR, risk ratio; CI, confidence interval.

## 4 Discussion

Although setting a target for blood pressure is a common treatment strategy for critically ill patients with shock, the target blood pressure for vasodilatory shock remains controversial. In addition, the optimal blood pressure control target according to the patient’s background, such as older age or a history of cardiovascular disease, has not been studied sufficiently.

To our best knowledge, this meta-analysis of three studies on optimal blood pressure control for vasodilatory shock allowed for the use of a large sample size, which was expected to be more representative of the patient population, leading to more accurate results. Also, it was meaningful to re-examine the subgroup analyses which showed conflicting results in previous RCTs.

This meta-analysis of three trials comparing a higher MAP target group with a lower MAP target group showed no significant difference in all-cause mortality. In the subgroup analysis for patients with chronic hypertension and even in patients aged ≥65 years, the intervention for the higher MAP target did not significantly improve mortality compared to the lower MAP target.

The results of the mortality rate comparing the higher and lower MAP groups were consistent in the three integrated trials (Asfar et al., 2014; Lamontagne et al., 2016; Lamontagne et al., 2020). There was little evidence that higher blood pressure control improves mortality rate. This was supported by the finding that there was no difference in mortality in the subgroup analysis of patients with chronic hypertension and older adults. Although there was no difference in serious adverse events associated with vasopressors between the two groups, the incidence of supraventricular arrhythmia was significantly higher in the higher MAP group. Refraining from excessive blood pressure control with vasopressors may reduce adverse events, such as arrhythmias. For these reasons, patient management with an MAP maintained at a value greater than 65 mmHg has not been recommended routinely in patients with vasodilatory shock. The Surviving Sepsis Campaign guideline 2021 also supported this result (Evans et al., 2021). However, there were no discussions with respect to personalized blood pressure control targets based on individual patient medical history, such as cardiovascular diseases and chronic hypertension.

With regard to the use of renal replacement therapy in patients with chronic hypertension, on the one hand, the SEPSISPAM (Asfar et al., 2014) trial showed a significantly lower use in the high MAP target group. On the other hand, the 65 trial (Lamontagne et al., 2020) showed no significant difference in renal replacement therapy between the high-and low-MAP target groups. Performing the integrated analysis of these two RCTs, the higher MAP target group showed lower use of renal replacement therapy in patients with chronic hypertension. A previous study reported that patients with chronic hypertension were required to have a higher MAP than those without chronic hypertension in order to maintain renal blood flow (Abuelo, 2007). Therefore, management of a higher MAP target may be helpful from the perspective of renal protection. Furthermore, the actual MAP in the higher target group of SEPSISPAM was the highest (85–90 mmHg) among the three RCTs, which may have been associated with a reduction in renal replacement therapy. In addition, a previous prospective study suggested that management with a higher MAP target of 72–82 mmHg was needed to avoid acute kidney insufficiency (AKI) (Badin et al., 2011). However, this finding was based on a subgroup analysis that needs to be interpreted carefully.

The present meta-analysis evaluated RCTs of vasodilatory shock patients; however, there may have been differences in the pathophysiology between septic shock and other vasodilatory shocks. Patients with septic shock generally demonstrate an increased oxygen demand due to systemic hypermetabolism, whereas a similar increase in oxygen demand does not occur in patients after general anesthesia or in patients with spinal cord injury. Thus, patients with septic shock may require more stringent blood pressure control to meet the oxygen demands of each organ. In this regard, it should be noted that, although the 65 trial (Lamontagne et al., 2020) included a large number of patients with vasodilatory shock, which may have affected the results of the present meta-analysis, the proportion of patients with septic shock in the trial was less than half. In terms of the optimal blood pressure target in septic shock patients with chronic hypertension, the results of an ongoing RCT (UMIN000041775) may provide further important insights into this topic.

This study had several limitations. First, the actual MAP in the lower MAP group exceeded the target blood pressure values defined in each trial, and each actual blood pressure value differed by trial. Second, we did not obtain sufficient data on mortality and secondary outcomes according to the specific subgroups in each trial. Therefore, we could not evaluate them individually according to the patient’s background. Finally, the evidence obtained in this analysis may be helpful for older adults, as 92.1% of patients enrolled in this analysis were over 65 years old; thus, the generalizability of our findings may be limited because of the relatively smaller proportion of younger patients.

The latest meta-analysis, which included an additional recent RCT, also showed no significant difference in all-cause mortality between the higher and lower MAP target groups. The results of most subgroup analyses also supported this conclusion, suggesting that higher MAP management not be recommended routinely for all vasodilatory shock patients. However, management of a higher MAP target may be beneficial in terms of reducing renal replacement therapy, only for patients with chronic hypertension. Further trials focusing on patients with chronic hypertension as well as older adults are needed.

## Data Availability

The original contributions presented in the study are included in the article/Supplementary Material, further inquiries can be directed to the corresponding author.

## Abbreviations

APACHE, Acute Physiology and Chronic Health Evaluation; IQR, interquartile range; ICU, intensive care unitMAP, mean arterial pressure; SAPS, simplified acute physiology score; SD, standard deviation.
